# Surveillance of Shrew-Borne Hantaviruses Expands Viral Host Range in Hungary

**DOI:** 10.3390/ani16020223

**Published:** 2026-01-12

**Authors:** Gréta Varga, Renáta Dóró, Anett Kuczmog, Győző Horváth, Gábor Kemenesi, Krisztián Bányai, Mónika Madai

**Affiliations:** 1 National Laboratory of Virology, Szentágothai Research Centre, University of Pécs, 7624 Pécs, Hungary; varga.greta@pte.hu (G.V.); kuczmog.anett@pte.hu (A.K.); kemenesi.gabor@pte.hu (G.K.); 2 Institute of Biology, Faculty of Sciences, University of Pécs, 7624 Pécs, Hungary; hgypte@gamma.ttk.pte.hu; 3 Institute of Physiology, Medical School, University of Pécs, 7624 Pécs, Hungary; doro.renata@pte.hu; 4 Department of Medical Biology, Medical School, University of Pécs, 7624 Pécs, Hungary; 5 Department of Pharmacology and Toxicology, University of Veterinary Medicine, 1078 Budapest, Hungary

**Keywords:** RT-PCR, virus monitoring, soricine hantaviruses

## Abstract

Since the late 2000s, a growing diversity of distinct hantaviruses has been identified in shrews across Europe. However, knowledge about shrew-borne hantaviruses in Hungary is limited. This study was initiated to investigate the prevalence and host range of these viruses in shrew populations in Hungary. Between 2007 and 2011, 129 shrew specimens, representing six species, were collected from four locations in Southern Transdanubia. Lung tissues were analyzed for the presence of hantaviral RNA by RT-PCR targeting the L genomic segment. Hantavirus RNA was detected in four shrew species. This study provides the first molecular evidence of hantavirus infection in the pygmy shrew (*Sorex minutus*), Miller’s water shrew (*Neomys milleri*), and the Eurasian water shrew (*Neomys fodiens*) in Hungary, while also confirming infection in the Eurasian common shrew (*Sorex araneus*). The overall prevalence varied among species, with marked detection rates in *S. minutus* (50%) and *N. fodiens* (33.3%). Our findings document the co-circulation of hantaviruses in four distinct shrew species within the same ecosystems in Hungary, thereby expanding the known host range for these viruses within the country. This contributes to the understanding of the complex hantavirus landscape in Central Europe and highlights the need for genetic characterization of identified viruses to assess their evolutionary dynamics.

## Simple Summary

Hantaviruses have been categorized as rodent-borne pathogens; however, it has been known for some time that other small animals, including shrews, can also carry these viruses. Understanding which animals carry these viruses is important for public health, but there has been little research on this topic in Hungary. This study aimed to find out how common hantaviruses are in different shrew species in Hungary. Over a five-year period, we found evidence of hantavirus infection in four different species: the Eurasian common shrew, the pygmy shrew, Miller’s water shrew, and the Eurasian water shrew. The findings show that several shrew species in Hungary carry hantaviruses and that these viruses circulate in different species living in the same area. This information is a crucial first step to assessing the potential animal and human health risks and understanding better how these viruses spread in the wild.

## 1. Introduction

Hantaviruses belong to the genus *Orthohantavirus* within the family *Hantaviridae* of the order *Elliovirales*. They are globally distributed, tri-segmented, negative-sense RNA viruses. Traditionally, hantaviruses have been recognized as rodent-borne pathogens; however, it is now well established that shrews and other small mammals also serve as natural hosts [1,2]. Human infections, acquired through the inhalation of aerosols from contaminated rodent excreta, can lead to severe, often lethal diseases [3]. The rodent-borne nature of orthohantaviruses has been challenged over the past two decades by the discovery of an extensive diversity of distinct hantaviruses in other small mammals, including bats, moles, and particularly shrews.

The first record of a shrew-associated hantavirus emerged with the isolation of Thottapalayam virus (TPMV) from a *Suncus murinus* (Linnaeus, 1766) in India in the 1960s [4]. Decades later, molecular techniques confirmed that TPMV is a shrew-borne hantavirus, phylogenetically distant from rodent-borne lineages [5]. This finding, along with long-ignored evidence of hantaviral antigens in *Sorex araneus* (Linnaeus, 1758) and *Neomys fodiens* (Pennant, 1771) from Russia and the Balkan peninsula, sparked enhanced interest and investigation [6,7]. A cornerstone in European hantavirus research was the first molecular identification of Seewis virus (SWSV) in a Eurasian common shrew (*S. araneus*) captured in Switzerland in 2006 [8]. This discovery definitively established certain widely distributed shrew species as hantavirus reservoirs in Europe.

Subsequent surveillance, including retrospective investigations carried out on archived *S. araneus* specimens, has revealed that SWSV is widespread across Eurasia, mirroring the vast geographic range of its primary host, the Eurasian common shrew. The SWSV, or, more precisely, the viral genomic RNA, has now been detected in *S. araneus* from numerous Northern and Central European countries. Investigations have shown that SWVS circulates in Siberia (Russia), as well [9,10,11,12]. While *S. araneus* is considered the principal reservoir, SWSV RNA has also been found in several other shrew species that share the same habitat. These include, e.g., the Eurasian pygmy shrew (*S. minutus* Linnaeus, 1766) in parts of Europe, as well as the tundra shrew (*Sorex tundrensis* Merriam, 1900), and the Siberian large-toothed shrew (*Sorex daphaenodon* Thomas, 1907) in Siberia [12]. Even some rodents have been reported to harbor SWSV [13]. These findings have prompted discussions about whether these detections represent “spillover” infections from the main host or indicate a broader, more complex host range for SWSV [14].

The landscape of shrew-borne hantavirus ecology and epizootiology in Europe was further diversified when other, genetically distinct viruses were discovered. Examples include Asikkala virus (ASIV), a pathogen with a fairly broad distribution from Finland to Central Europe, whose putative primary host is *S. minutus* [15]. In Poland, Boginia virus (BOGV) has been identified in the Eurasian water shrew (*N. fodiens*) [16]. Studies have documented the co-circulation of these different hantaviruses in their respective hosts within the same geographical areas, creating opportunities for complex ecological interactions like host-sharing and potential viral genetic exchange [17,18].

This study aimed to assess the prevalence and host range of shrew-borne hantaviruses in Hungary through molecular surveillance of multiple shrew species. Hungary was originally placed on the map of shrew-borne hantavirus research by American researchers [9,19]. Building on these earlier observations, the present study, initiated by ecologists and virologists based in Hungary, was conducted over five years. The findings of this survey expand the range of soricine host species currently known to harbor hantaviruses in Hungary and place the national data into a broader European epidemiological context, providing a basis for future genetic and evolutionary studies.

## 2. Materials and Methods

### 2.1. Sample Collection and Processing

**Figure 1:**
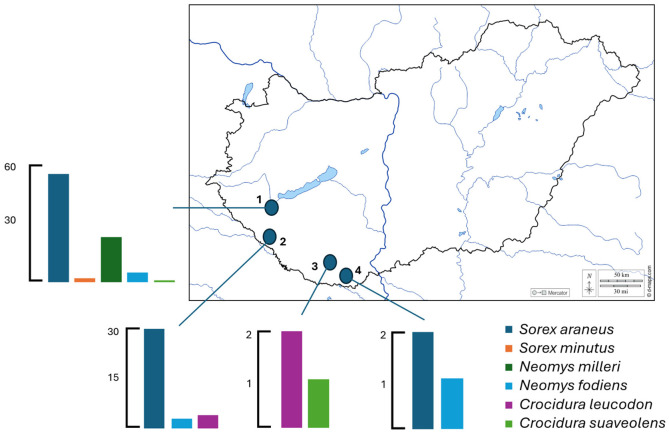
Number of shrew species collected at the four study areas (1. Kis-Balaton, 2. Gyékényes, 3. Gyód, 4. Matty).

Animals were collected between 2007 and 2011 using live traps at four locations in Southern Transdanubia (Kis-Balaton, Gyékényes, Gyód, and Matty; Figure 1). Trapping and handling of animals were authorized by the National Inspectorate for Environment, Nature Conservation and Water Management, Hungary (permit no. 14/6044-2/2010). The sampling sites represented distinct habitat types: Gyód was an agricultural area, Matty was a lakeshore mosaic habitat, Gyékényes was a forest habitat, and Kis-Balaton was characterized by extensive stands of tall sedge and reedbeds.

Specimens that died of natural causes within the traps were used for further investigations. Key data (species name, collection site, time, habitat type, sex, body weight, age) were recorded and entered into a computer database (Microsoft Access, MS Office 365). Taxonomic classification was performed by a qualified taxonomist. Following transport to the laboratory, the animals were stored at −20 °C until the laboratory examinations began.

Specimens from several insectivore species were used: *S. araneus*, *S. minutus*, *Neomys milleri* (Mottaz, 1907), *N. fodiens*, *Crocidura leucodon* (Hermann, 1780), and *Crocidura suaveolens* (Pallas, 1811). It should be noted that before 2015, *Neomys milleri* was commonly referred to as *Neomys anomalus* (Cabrera, 1907) in the literature. Subsequent taxonomic revisions, supported by morphological and molecular data, have led to the recognition of *N. milleri* as a separate species in more recent classifications. Although the trapping was conducted before the taxonomic revision, throughout this study, we refer to *Neomys anomalus* as *Neomys milleri*, in accordance with the currently accepted taxonomic classification [20,21].

During dissection, the brain and lungs were removed and placed in Eppendorf tubes, then stored at −80 °C. For molecular studies, lung tissue samples were thawed and homogenized in 500 μL 1 × PBS using a Minilys homogenizer (Bertin Instruments, Montigny-le-Bretonneux, France) with one glass bead (2.5–2.8 mm). The samples were vortexed, then centrifuged for 10 min at 14,000× *g* (Hermle Z 233 MK-2, Hermle Labortchnik, Wehingen, Germany) at 4 °C. The resulting supernatant was used for RNA extraction.

### 2.2. Viral RNA Extraction

Nucleic acid purification was performed using the TRIzol^®^ (Invitrogen, Carlsbad, CA, USA) method according to the manufacturer’s protocol. Briefly, 150 μL of the prepared supernatant was added to 500 μL of TRIzol^®^ reagent. The mixture was vortexed for 30 s and incubated for 5 min at room temperature. After a brief centrifugation to collect droplets, 100 μL of chloroform was added, followed by vortexing and a 5 min incubation at room temperature. The mixture was then centrifuged for 15 min at 14,000× *g* at 4 °C. The upper aqueous phase (approx. 400 μL) was transferred to a new tube containing 400 μL of isopropanol to precipitate the RNA, and the samples were incubated for 1 h at −20 °C. After incubation, the tubes were centrifuged for 15 min at 14,000× *g* at 4 °C. The isopropanol was removed, and the nucleic acid pellet was washed with 500 μL of 70% ethanol, followed by a 5 min centrifugation at 14,000× *g* at 4 °C. After removing the ethanol, the pellets were air-dried for 15 min. Finally, the RNA was redissolved in 35 μL of nuclease-free water (Promega, Madison, WI, USA) and stored at −80 °C.

### 2.3. Reverse Transcription Polymerase Chain Reaction (RT-PCR)

Target gene amplification was performed with the Qiagen OneStep RT-PCR Kit (Qiagen, Hilden, Germany), following the manufacturer’s instructions and using 25 pmol each of the gene-specific primers, Hanta L_2_rev and Hanta L_2_fw [22]. The amplification program was as follows: reverse transcription at 50 °C for 30 min; PCR enzyme activation at 95 °C for 15 min; 40 cycles of amplification starting with denaturation at 94 °C for 1 min and followed by annealing at 54 °C for 45 s and extension at 72 °C for 1 min; and a final extension at 72 °C for 10 min. The resulting products were stored at −20 °C. The PCR products were analyzed by agarose gel electrophoresis using a 2% agarose gel with TBE buffer and GRgreen stain (Labgene Scientific, Châtel-Saint-Denis, Switzerland). The gel was run at a constant 90 V for 60–70 min.

### 2.4. Gel Extraction and Sequencing

Bands of the expected size were manually excised from the agarose gel under UV illumination. The DNA was purified from the gel slices using the QIAquick Gel Extraction Kit (Qiagen, Cat. no. 28706), following the manufacturer’s protocol precisely. Purified samples were stored at −20 °C.

For the sequencing reaction, a cycle sequencing PCR was performed in a 10 μL volume containing the purified nucleic acid (20–25 pmol), 10 pmol of a single primer, and the BigDye Terminator v1.1 Cycle Sequencing Kit (Applied Biosystems, Waltham, MA, USA). The thermal profile was: 96 °C for 1 min, followed by 25 cycles of 96 °C for 20 s, 50 °C for 5 s, and 60 °C for 4 min. The dye-labeled products were purified using sodium acetate/ethanol precipitation. After being washed with 70% ethanol, the DNA pellet was dried and resuspended in Hi-Di Formamide. The products were run on an ABI PRISM 310 Genetic Analyzer (Applied Biosystems™, Waltham, MA, USA).

Nucleic acid sequences showing the most closely related homology were identified in GenBank through BLAST (version: 2.15.0) searches [23].

### 2.5. Literature Search

A structured literature search was conducted to place the results of the present study into a broader European context of shrew-borne hantavirus surveillance. The primary database used was PubMed, selected for its comprehensive coverage of peer-reviewed biomedical and virological literature. In addition, ResearchGate was included to identify relevant articles, preprints, and author-shared publications that may not yet be indexed in PubMed, particularly older regional studies and surveillance reports.

Searches were performed using combinations of the following keywords: *hantavirus*, *orthohantavirus*, *shrew*, *Sorex*, *Neomys*, *Crocidura*, *soricid*, *insectivore*, *surveillance*, *prevalence*, and *Europe*. Boolean operators were applied where appropriate (“hantavirus AND shrew”, “Sorex AND hantavirus”, “soricid-borne hantavirus”).

Studies were included if they reported molecular or virological evidence of hantavirus infection in shrews from European countries. From the selected publications, data were manually extracted on country of study, sampling period, shrew species examined, diagnostic methods used, hantavirus detection rates, and available viral genetic information. Review articles were used to identify additional primary studies through reference screening.

## 3. Results

### 3.1. PCR Detection of Hantavirus Infection in Hungarian Shrews

We processed 129 shrew samples collected between 2007 and 2011 at four locations (Kis-Balaton, Gyékényes, Gyód, and Matty) that lie in the Transdanubian region south of lake Balaton. The shrews that died in the traps were individuals of the following six species: *S. araneus* (*n* = 87), *S. minutus* (*n* = 2), *N. milleri* (*n* = 23), *N. fodiens* (*n* = 9), *C. leucodon* (*n* = 2), and *C. suaveolens* (*n* = 6) (Figure 1). These six species represent all but one of the seven shrew species known to occur in Hungary, with the Alpine shrew (*Sorex alpinus* Schinz, 1837) being the only species not captured during the study period.

Table 1 summarizes the host species-specific viral detections at the four sampling sites. In brief, hantaviral RNA could not be amplified from samples prepared from *C. suaveolens* and *C. leucodon*. In contrast, hantavirus was detected in several samples from the other four shrew species. In the Kis-Balaton region, 10 out of 86 specimens (11.6%) tested positive for hantaviral RNA; positive samples were collected from *S. araneus* (*n* = 6, 10.9%), *S. minutus* (*n* = 1, 50%), *N. milleri* (*n* = 1, 4.3%) and *N. fodiens* (*n* = 2, 40%). The combined virus detection rate was somewhat lower in the Gyékényes region (3 out of 37, 8.1%), with positive samples taken from *S. araneus* (*n* = 2, 6.7%) and *N. fodiens* (*n* = 1, 33.3%).

### 3.2. Sequence Analysis of L-Segment PCR Amplicons

The BLAST analysis of the obtained L-segment PCR amplicons showed the highest similarity to available hantavirus sequences. However, the amplified ~170 bp fragment represents a highly conserved region of the hantavirus RNA-dependent RNA polymerase gene, where limited sequence variability is expected across different hantavirus species. Due to the short length of the obtained sequences, further sequencing and phylogenetic analyses were not feasible, precluding reliable virus species assignment.

### 3.3. Inclusion of Soricine Hantavirus Surveillance Data in a European Framework

To place our surveillance data into the context of shrew-borne hantaviruses in Europe, we collected relevant literature and extracted data from those studies. This analysis of hantavirus prevalence in shrew populations across several countries in Northern and Central Europe revealed clear geographical and host-species-specific variations. The studies reported that individuals of eight shrew host species were trapped and tested (*S. araneus*, *S. minutus*, *Sorex coronatus* (Millet, 1828), *S. alpinus*, *N. fodiens*, *N. milleri*, *C. leucodon*, and *C. suavolens*), out of which only four species gave positive test results with hantavirus RT-PCR or viral metagenomics. These were *S. araneus*, *S. minutus*, *N. fodiens*, and *N. milleri*.

Studies differed in country-specific sample size. For example, research groups in Switzerland, Slovakia, and Croatia each processed 10 samples or fewer, whereas colleagues in Poland processed over 460 samples combined across four studies. There were substantial fluctuations in hantavirus prevalence, not only between different countries and time periods but also across various locations within the same country. Despite epidemiologic monitoring covering a considerable period between the 1980s and the 2010s, the total number of hantavirus-positive shrews reported in Europe remains close to, but below, 200.

Taking into account all these limitations, high hantavirus detection rates were recorded for *S. araneus* in Czechia (75%) from 2017 to 2020, and lower in another study (40.3%) from 2003 to 2010. Similarly, a study on *S. araneus* in Finland recorded a prevalence of 54.5% for SWSV. In contrast, some countries showed consistently lower detection rates of hantavirus infection in shrews. For example, studies in Sweden reported prevalence in *S. araneus* at 1.5% and 11.8% in different periods. Germany found a low rate of 2.5% in both *S. araneus* and *S. minutus*. Poland presents a more complex picture with highly variable results for *S. araneus*, ranging from as low as 0.8% to as high as 30% across different study periods. The data from Hungary is also noteworthy. An earlier study, which processed samples from 1997 to 2000, found a 19.7% prevalence in *S. araneus*, and this rate appeared to decrease to 9.2% in the current study reporting data from 2007 to 2011. In addition, these new data from Hungary uncovered high viral prevalence even in less studied hosts, such as *S. minutus* (50%) and *N. fodiens* (33.3%) (Table 2).

## 4. Discussion

This study confirms that *S. araneus*, a widespread European soricine species, harbors hantaviruses and provides the first molecular evidence of hantavirus infection in *S. minutus*, *N. milleri*, and *N. fodiens* in Hungary, thereby expanding the known host range for these viruses within the country [9,19]. Our survey revealed a hantavirus prevalence of 9.2% in *S. araneus*, one of the most commonly investigated shrew reservoir species in Europe. This rate is lower than the 19.7% previously reported in Hungary from samples collected between 1997 and 2000, but it falls within the wide prevalence range observed across the continent, which ranges from less than 1% in Poland to 75% in Czechia [17,28]. These variations may be associated with spatiotemporal heterogeneity observed in hantavirus occurrence, which can vary across locations and sampling periods.

While SWSV is the most frequently identified lineage of shrew-borne hantaviruses that is typically associated with *S. araneus* across its vast geographic range, the lack of sequence data in our study prevents its definitive confirmation. In addition to *S. araneus*, which is the most important host for SWSV, the virus was reported to occur in *N. milleri* in Poland, Croatia, and Austria [24,28], as well as in *S. minutus* in Czechia and Germany [25]. Regarding other European soricine hantaviruses, such as ASIV, BOGV, and Altai virus (ALTV, genus *Mobatvirus*), reporting has been scarce compared to those for SWSV. For example, ASIV was reported to occur in *S. minutus* and *S. araneus* in Czechia [15,17], BOGV was identified in *N. fodiens* in Poland [28], ALTV was recorded in *S. minutus* in Poland, and in *S. araneus* in Sweden, Hungary, and Finland [18,19,28,30]. In addition, some rodent species, including yellow-necked mouse (*Apodemus flavicollis*) and wood mouse (*Apodemus sylvaticus*), were reported to carry SWSV in Czechia [13].

An intriguing observation was the 50% detection rate of hantaviral RNA in *S. minutus*, the putative host for ASIV. Although our finding is based on a very limited sample size (one positive out of two animals tested), it sharply contrasts with the lower prevalence reported in Germany (2.5%) and Poland (3.8%) [15,16,25,26]. This preliminary but high prevalence suggests that *S. minutus* could be a potentially important hantavirus reservoir in Hungary. Similarly, the detection rates of hantaviral RNA in *N. fodiens* (33.3–40%) aligns with findings from Poland (25%), where it is the recognized host of BOGV [16,26], but contrast with other studies from different countries where the virus was absent. The detection of hantavirus RNA in *N. milleri* (4.3%) is also the first record for Hungary, adding valuable data for this less-studied shrew species, which has shown higher hantavirus prevalence elsewhere, e.g., in Croatia (25%), although based on a limited sample size [24]. Collectively, while these findings provide the first molecular evidence of hantavirus infection for these host species in Hungary, the small number of specimens captured limits the statistical robustness and representativeness of these estimates. Consequently, these percentages should be viewed as preliminary indicators of viral presence rather than definitive population-level prevalence.

The main limitation of this study was the failure to amplify and sequence the viral S genomic segment, which prevented the classification of the detected hantaviruses. While our PCR assay based on the L genomic segment was suitable for confirming the presence of hantaviral RNA in shrew tissue samples, the absence of sequence data required for viral classification leaves critical ecological and evolutionary questions unanswered. We could not determine if the detected viruses were the expected SWSV, ASIV, ALTV, and BOGV in their respective hosts or if they represent spillover events from cohabiting heterologous host species. Furthermore, the potential for identifying novel viral lineages was not realized either. Overall, unraveling these genetic details of circulating soricine hantaviruses in Hungary would be essential for understanding virus–host relationships and the evolutionary dynamics that shape viral diversity.

Despite these limitations, our findings document the co-circulation of hantaviruses in four distinct shrew species within the same ecosystems in Hungary. This contributes to the growing understanding of the complex hantavirus landscape in Central Europe and highlights the need for continued surveillance.

## 5. Conclusions

The pathogenic potential of shrew-borne hantaviruses for humans, domestic animals, and their natural hosts remains undetermined. While human exposure likely occurs through the inhalation of aerosolized excreta—similar to the transmission routes of well-documented rodent-borne hantaviruses—the frequency of human-shrew contact is generally lower than contact with synanthropic rodents. However, the documented co-circulation of these viruses within diverse Hungarian ecosystems, ranging from agricultural areas to reedbeds, suggests that specific occupational or recreational activities could still facilitate transmission. Consequently, the wide geographic distribution and genetic diversity of these viruses underscore the necessity of expanding surveillance to include soricine hosts; this information is crucial for assessing potential public health risks and is essential for a proactive “One Health” approach to emerging zoonoses.

## Figures and Tables

**Table 1:** Detection rates of hantaviral RNA in different shrew species collected at four locations between 2007 and 2011.

Area of Collection	No. of Animals	Species	Tested Animals	Positive by PCR (%)
Kis-Balaton	86	*Sorex araneus*	55	6 (10.9)
		*Sorex minutus*	2	1 (50)
		*Crocidura suaveolens*	1	0
		*Neomys fodiens*	5	2 (40)
		*Neomys milleri*	23	1 (4.3)
Gyékényes	37	*Sorex araneus*	30	2 (6.7)
		*Crocidura leucodon*	4	0
		*Neomys fodiens*	3	1 (33.3)
Gyód	3	*Crocidura leucodon*	2	0
		*Crocidura suaveolens*	1	0
Matty	3	*Sorex araneus*	2	0
		*Neomys fodiens*	1	0
**Total**			**129**	**13 (10)**

**Table 2:** Comparative prevalence of hantaviruses in European shrew species reported in published studies and this study. (%: proportion of hantavirus-positive animals within a given species; *n*: number of positive/total number of tested individuals).

Country	Collection Period	*S. araneus*		*S. minutus*		*N. fodiens*		*N.* *milleri*		References
		n	%	n	%	n	%	n	%	
Croatia	2003–2017	1/2	50	0/1		0/1	0	1/4	25	[24]
Czechia	2017–2020	3/4	75	0/2	0	0/2	0			[17]
	2003–2010	42/104	40.3		15.7					[25]
Germany	1983–1985, 2002–2010	6/239	2.5		2.5					[15,25]
Finland	1982	12/22	54.5							[9]
	2013–2014	42/225	18.6							[10]
Hungary	1997, 1999–2000	13/66	19.7							[9]
	2007–2011	8/87	9.2	3/9	50	3/9	33.3	1/23 *	4.3	this study
Poland	2010–2013	14/53	26.4	4/16	3.8	4/16	25	1/12 *	8.3	[16,26]
	2009–2012, 2014–2015	3/10	30					0/1 *	0	[27]
	1990–2009, 2013–2017	1/117	0.8	0/72	1.5	0/72	0	0/65 *	0	[28]
Slovakia	2008–2011	2/20	10							[25]
Slovenia	1990–2006	7/38	18.4	0/8		0/8	0	0/13 *	0	[29]
Sweden	1998	3/198	1.5							[11]
	2015–2017	13/110	11.8		**					[18]
Switzerland	2006	1/5	20					0/1 *	0	[8]

* The authors used the taxon name *N. anomalus*. ** The authors reported that more than two dozen *S. minutus* were also trapped, but the virus diagnostic results were not disclosed.

## Data Availability

The original contributions presented in this study are included in the article. Further inquiries can be directed to the corresponding authors.
